# A randomized pilot study evaluating socially assistive robot effects on patient engagement and care quality

**DOI:** 10.1038/s41746-025-02117-9

**Published:** 2025-12-02

**Authors:** Izidor Mlakar, Urška Smrke, Valentino Šafran, Igor Robert Roj, Bojan Ilijevec, Samo Horvat, Vojko Flis, Nejc Plohl

**Affiliations:** 1 Faculty of Electrical Engineering and Computer Science, University of Maribor, Maribor, Slovenia; 2 University Medical Center Maribor, Maribor, Slovenia; 3 Faculty of Arts, Department of Psychology, University of Maribor, Maribor, Slovenia

**Keywords:** Health care, Randomized controlled trials, Preclinical research, Computational science, Human behaviour

## Abstract

Healthcare faces significant challenges, including workforce shortages and increasing demands. Socially assistive robots (SARs) have emerged as potential solutions to augment care, but their implementation in hospital wards remains largely unexplored. We conducted a randomized external pilot study (ISRCTN Registry, ISRCTN96689284, registered 24/02/2022) evaluating SAR intervention feasibility and effects on patient engagement and perceived quality of care (co-primary outcomes) and health-related quality of life (secondary outcome) in surgical wards. Patients (*N* = 229) at University Medical Center Maribor were allocated to SAR intervention (standard care + SAR) or control groups (standard care only). The SAR utilized validated, story-driven conversational capabilities providing standardized patient education, support, and basic triage through predefined dialog flows. While overall effects on patient engagement and perceived care quality were limited, the intervention showed positive impact on pain management. Contextual factors moderated intervention effects, highlighting SAR potential in specific domains. No substantial negative effects were detected. High retention rates demonstrated practical feasibility of SAR implementation in surgical settings.

## Introduction

The healthcare sector is facing significant challenges, including workforce shortages and increasing demands, due to population aging^1^. Europe alone is faced with a shortage of approximately 2 million healthcare professionals^2^. These issues can lead to higher workloads for healthcare staff, potentially resulting in burnout, reduced job satisfaction, and suboptimal patient care^3,4^. Moreover, there has been a paradigm shift in healthcare objectives, with active patient engagement and self-management emerging as primary goals in the 21st century^5,6^. Active patient engagement refers to the process of involving patients in their own care, encouraging them to participate in decision-making and take responsibility for their health outcomes^7^. Self-management, related closely to engagement, involves patients developing the skills and confidence to manage their health conditions effectively^8^. These concepts have gained prominence due to their potential to improve health outcomes, enhance patient satisfaction, contribute to a higher health-related quality of life, and reduce healthcare costs^9–12^.

However, achieving active patient engagement and promoting self-management can be challenging^13,14^, particularly in the context of the workforce shortages and time constraints faced by healthcare providers^15^. Innovative solutions are essential to complement traditional approaches and support both healthcare providers and patients in achieving better health outcomes. Socially assistive robots (SARs) have emerged as a promising technology that could, potentially, address these challenges. These robots are designed to provide assistance through social interaction, offering services such as education, feedback, support, coaching, and companionship^16,17^. The SARs are equipped with multimodal sensing capabilities, allowing them to collect a wide array of data that can be used for personalizing healthcare and responding to individual patient needs^18–23^. The potential benefits of SARs in healthcare are multifaceted, particularly in promoting patient engagement and self-management. For patients, the SARs are hypothesized to enhance patient engagement through interactive education and continuous availability, while simultaneously improving the perceived quality of care by providing consistent, personalized support that complements human-delivered care^16,17,24,25^. Namely, they can provide continuous support and education without the time constraints faced by human staff, potentially improving health literacy and patient empowerment^5^. This constant availability could be particularly beneficial in supporting patients’ self-management efforts, providing reminders, encouragement, and information as needed^24,25^. A recent study has also demonstrated that SARs may exert a positive effect on health-related quality of life in some contexts, such as in the case of socially isolated older adults^26^. For healthcare providers, the SARs may help alleviate their workload by assisting with tasks such as providing information, reminding about medication, and offering basic care instructions^27–29^.

Despite these potential advantages, the large-scale implementation of SARs in healthcare settings is progressing slowly^30^. The main barriers include ethical and workload-related concerns, driven further by education and technology expectations^31,32^. This may be extended further, in part, to a limited number of systematic studies conducted in real-life healthcare environments^18^. While existing research shows promise, many studies have focused on specific populations, such as the elderly with dementia or children with neurodevelopmental disorders^19,33–35^. Moreover, in the context of surgery wards, where patient engagement and adherence to post-operative instructions are crucial for recovery, the potential of SARs remains largely unexplored. Finally, there has been limited integration of SAR interventions within existing hospital care pathways in previous research^36,37^.

To address the limited evidence on SAR acceptance in surgical settings, our research team previously conducted a comprehensive acceptability study, examining attitudes and expectations toward socially assistive humanoid robots, among both patients and healthcare professionals in surgical wards^32^. This preliminary work revealed generally positive attitudes toward SAR technology, with patients showing moderate to high acceptance levels. However, the acceptability study also identified important individual differences and contextual factors related to SAR acceptance, such as age and prior technology experience. These findings highlighted the need for empirical evaluation of actual SAR implementation, to determine whether positive pre-implementation attitudes translate into meaningful patient outcomes during real-world deployment. The acceptability study informed our intervention design and outcome selection, particularly the inclusion of robot acceptance as a potential moderator of intervention effectiveness.

Overall, there is a clear need for more robust studies on the use of SARs in healthcare settings. Our study explores how a SAR can be used to deliver gamified nursing education and physiotherapy, complementing standard care practices in a surgical ward. The approach provides valuable insights into how SARs can be integrated effectively into existing healthcare workflows. By providing evidence on the feasibility and potential impacts of SAR implementation on patient outcomes, our study makes a significant contribution to the field, and paves the way for future definitive trials.

Building on previous studies and our prior acceptability research that implied the feasibility of SAR deployment and identified key moderating factors^32^, the present study represents the next logical step in our research program by evaluating the actual implementation effects on patient outcomes. The primary aim of the present study was to assess the feasibility and potential effects of implementing a SAR intervention in surgery wards. We selected patient engagement and perceived quality of care as the co-primary outcomes based on: (1) their theoretical centrality to SARs interventions; (2) their established relationships with important healthcare outcomes, including treatment adherence, patient satisfaction, and clinical effectiveness; (3) stakeholder input from co-creation workshops conducted during the HosmartAI project^38^; and (4) the availability of validated, psychometrically sound instruments for measuring both constructs^5,6,9–12^. Namely, patient engagement reflects the active participation and self-management behaviors that SARs are designed specifically to promote through interactive education and continuous support availability^7,24,25^. The SAR intervention may enhance patient engagement through several specific mechanisms. The first one is interactive education delivery. Specifically, the robot may provide multimodal educational content about wound care, physiotherapy, and self-management that promotes active learning and retention^39,40^. Second, another important mechanism may be continuous availability; unlike human staff constrained by time and workload, the SAR provides consistent access to information and support, facilitating ongoing patient participation in care decisions^16,41^. Lastly, SARs have the advantage of providing personalized feedback. In other words, the robot may adapt interactions based on patient responses, which may, in turn, promote individualized engagement and self-efficacy development^42^.

Perceived quality of care captures patients’ subjective evaluation of their overall care experience, which may be enhanced through the consistent, personalized support that SAR interventions provide as a complement to human-delivered care^16,17^. The SAR intervention may influence the perceived quality of care through various pathways, for example, by providing additional educational and support interactions beyond what staff time allows, potentially enhancing patients’ perception of comprehensive care^42–44^. Moreover, additional potential benefits are the consistency of information (i.e., standardized, evidence-based content delivery may improve patients’ confidence in the care quality) and novelty (i.e., the innovative nature of robot-assisted care may influence patient perceptions positively, though this could also create expectation-reality gaps)^16,45^.

Both domains are independently necessary to establish meaningful clinical benefit since (1) patient engagement without quality improvement would indicate technology acceptance but not therapeutic value, and (2) perceived quality of care without active engagement would suggest passive satisfaction but not the therapeutic alliance, essential for robotic intervention success. Additionally, health-related quality of life served as a secondary outcome, to capture broader impacts of the intervention on patient well-being. Health-related quality of life (HRQoL) is frequently included as a secondary outcome in studies of SAR interventions, aiming to assess the broader impact of the intervention on overall patient well-being^16,24^.

The specific objectives are as follows:

1. To assess the effect of the SAR intervention on patient engagement (co-primary outcome) compared with standard care.
2. To assess the effect of the SAR intervention on patients’ perceived quality of care (co-primary outcome) compared with standard care.
3. To assess the effect of the SAR intervention on patients’ health-related quality of life (secondary outcome) compared with standard care.
4. To evaluate the feasibility of implementing the SAR intervention in surgical wards by comparing retention rates between intervention and control groups, and determining intervention completion rates among the participants allocated to the SAR intervention.
5. To explore the potential moderators that impact the effectiveness of the SAR intervention on patient-related outcomes.
6. To estimate effect sizes for the co-primary outcomes, to inform the sample size calculations for future controlled trials.

## Results

### Sample characteristics

In total, 229 surgery patients were recruited for the study, an estimated 10% retention rate was used as a stop condition; 111 patients (48.5%) were allocated to the control group, whereas the remaining 118 patients (51.5%) were allocated to the intervention group. Out of the 229 recruited, only 206 patients provided their demographic data and completed the baseline measure partially (control group: *n* = 111, 53.9%; intervention group: *n* = 95; 46.1%). Of these, 133 (64.6%) were treated at the thoracic surgery department, and 73 (35.4%) at the abdominal and general surgery department. Most (*n* = 117; 56.8%) were female, while the rest (*n* = 89; 43.2%) identified as male during the study. Their age ranged from 20 to 85 years (*M* = 61.20, SD = 13.80). The most frequent procedures included hernia repair (control group: *n* = 14; 13.6%; intervention group: *n* = 10; 10.5%), gallbladder surgery (control group: *n* = 14, 12.6%; intervention group: *n* = 13, 13.7%), CT-guided punctures (control group: *n* = 20, 18.0%; intervention group: *n* = 22, 23.2%), and thyroid (control group: *n* = 15, 13.5%, intervention group: *n* = 19, 20.0%). In terms of education, the majority of the patients had finished lower (*n* = 64; 31.3%) or higher (*n* = 58; 28.2%) secondary education, followed by a Bachelor’s degree or equivalent (*n* = 41; 19.9%), primary school or less (*n* = 27; 13.1%); Master’s degree or equivalent (*n* = 11; 5.3%), and Doctoral degree or equivalent (*n* = 5; 2.4%). The control and intervention group did not differ based on the department they were admitted to (*χ*^2^(1) = 0.606, *p* = 0.467), gender (*χ*^2^(1) = 0.305, *p* = 0.672), education (*U* = 5124.50, *Z* = −0.14, *p* = 0.888), nor age (*t*(204) = 0.29, *p* = 0.770, *d* = 0.04), providing support for the demographic equivalence of the two groups. The baseline patient characteristics are summarized in Table 1.

**Table 1:** Baseline patient characteristics by group

	Control group (*n* = 111)	Intervention group (*n* = 95)	Total (*N* = 206)
Age, mean (SD)	61.46 (14.81)	60.89 (12.58)	61.20 (13.80)
Range	20-85	22-81	20-85
Gender, *n* (%)			
Female	65 (58.6)	52 (54.7)	117 (56.8)
Male	46 (41.4)	43 (45.3)	89 (43.2)
Education level, *n* (%)			
Primary school or less	16 (14.4)	11 (11.6)	27 (13.1)
Lower secondary education	35 (31.5)	29 (30.5)	64 (31.1)
Higher secondary education	28 (25.2)	30 (31.6)	58 (28.2)
Bachelor’s degree or equivalent	21 (18.9)	20 (21.1)	41 (19.9)
Master’s degree or equivalent	8 (7.2)	3 (3.2)	11 (5.3)
Doctoral degree or equivalent	3 (2.7)	2 (2.1)	5 (2.4)
Department, *n* (%)			
Thoracic surgery	69 (62.2)	64 (67.4)	133 (64.6)
Abdominal and general surgery	42 (37.8)	31 (32.6)	73 (35.4)

**Fig. 1:**
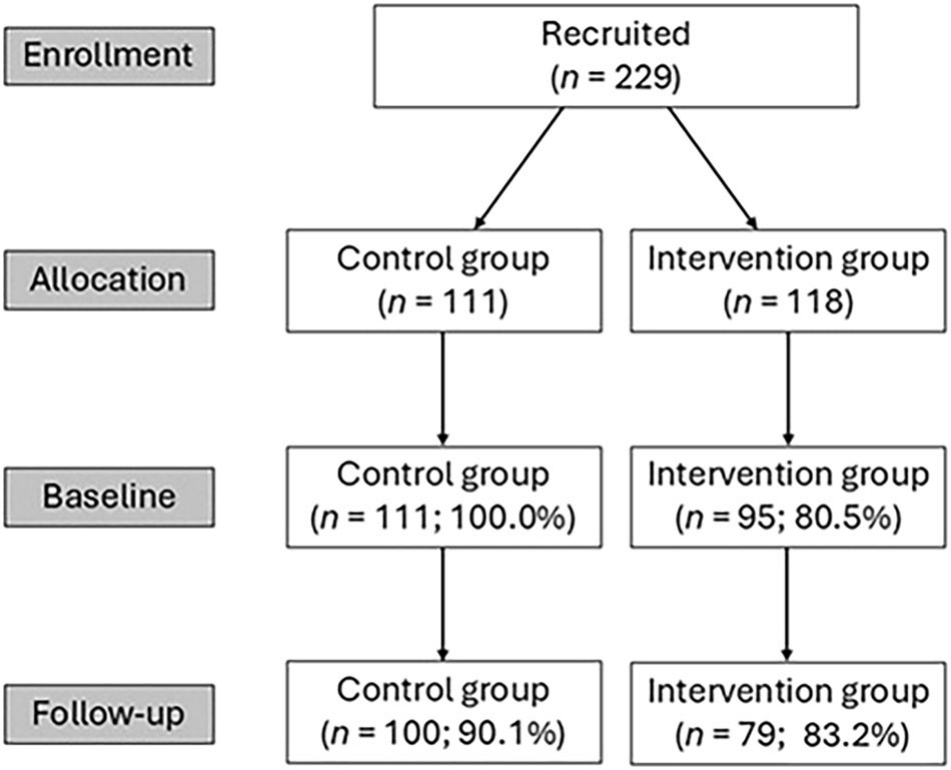
The flow chart of participants. The reasons for study discontinuation were documented and categorized into five main types. The largest share of participants (*n* = 23) submitted only partially completed questionnaires, or failed to complete the required baseline or final assessments, making their data unusable for analysis. This was followed by the participants who refused explicitly to participate further, or refused specific study procedures (*n* = 12), with the majority of the intervention group dropouts declining the use of digital health monitors, sometimes linked to postoperative pain or worsening health status. Furthermore, seven patients were transferred to another department, had procedures rescheduled, or were otherwise relocated, or, in rare cases, died during the study. The remaining patients dropped out because they felt unwell or uncomfortable with the intervention, leading to refusal after their initial exposure (*n* = 3), or dropped out due to ambiguous or unclear reasons (*n* = 5).

179 surgery patients (86.9% of those who completed the baseline measure) at least partly filled out the post-intervention measure, 100 from the control group (55.9%) and 79 from the intervention group (44.1%). Those who filled out the T2 measure and those who dropped out did not differ in terms of their group (*χ*^2^(1) = 2.16, *p* = 0.153), gender (*χ*^2^(1) = 0.08, *p* = 0.837), education (*U* = 2374.50, *Z* = −0.15, *p* = 0.881), and age (*t*(204) = −0.43, *p* = 0.669), indicating the absence of selective dropout based on the individuals’ group allocation and demographic variables. However, those who dropped out and those who did not differed in terms of the department they were admitted to (*χ*^2^(1) = 5.50, *p* = 0.029); specifically, while only 9.0% (*n* = 12) of thoracic surgery patients discontinued their participation, this was the case for 20.5% (*n* = 15) of the abdominal and general surgery patients. The flow chart of participants can be seen in Fig. 1 below.

No harms or unintended effects were observed in either control or intervention groups or were the cause of the dropout.

### Descriptive statistics and bivariate associations

We first calculated the basic descriptive statistics and bivariate associations for the whole sample, regardless of the individuals’ group allocation. The results are displayed in Table 2 below. Of particular importance to the present study, the results show weak to moderate positive associations between all the outcomes (patient engagement, perceived quality of medical care, health-related quality of life, and self-rated health) at the baseline and at follow-up; all the bivariate associations between the outcomes, bar the associations between the baseline perceived quality of care and baseline health-related quality of life indicators, were statistically significant.

**Table 2:** Descriptive statistics and bivariate associations

	*N*	*M*	SD	1	2	3	4	5	6	7	8	9	10	11	12
1. Gender	206	1.43	0.50	-											
2. Age	206	61.20	13.80	0.11	-										
3. Education	206	2.81	1.20	−0.11	−0.26^***^	-									
4. Department	206	1.35	0.48	0.11	−0.17^*^	0.15^*^	-								
5. Acceptance of robots	172	0.72	0.39	0.05	−0.07	0.13	0.15	(0.89)							
6. Patient engagement (T1)	198	4.31	0.59	0.14	−0.05	0.08	0.19^**^	0.15	(0.91)						
7. Patient engagement (T2)	174	4.26	0.59	0.15*	−0.09	0.03	−0.08	0.00	0.30^***^	(0.93)					
8. Perceived quality of care (T1)	195	6.30	0.95	0.01	0.06	0.02	−0.03	−0.03	0.25^***^	0.08	(0.82)				
9. Perceived quality of care (T2)	177	5.91	1.28	0.03	0.01	0.01	−0.29^***^	−0.06	−0.03	0.45^***^	0.14	(0.90)			
10. Health-related quality of life (T1)	206	2.67	0.30	0.00	−0.15*	0.15^*^	0.06	−0.01	0.33^***^	0.29^***^	0.12	0.07	(0.67)		
11. Health-related quality of life (T2)	172	2.67	0.33	−0.01	−0.06	0.08	−0.24^***^	−0.08	0.11	0.34^***^	0.11	0.35^***^	0.48^***^	(0.76)	
12. Self-rated health (T1)	206	68.50	17.95	0.08	−0.19^**^	0.11	0.03	0.01	0.39^***^	0.34^***^	0.10	0.15^*^	0.39^***^	0.23^**^	-
13. Self-rated health (T2)	172	72.12	19.00	0.09	−0.07	0.07	−0.14	0.07	0.24^**^	0.39^***^	−0.02	0.30^***^	0.31^***^	0.42^***^	0.38^***^

*Notes*. Gender: 1 = Female, 2 = Male. Department: 1 = Thoracic surgery, 2 = Abdominal and general surgery. Cases were excluded pairwise. ^*^p < 0.050, ^**^ *p* < 0.010, ^***^ *p* < 0.001.

### Effects on patient engagement and perceived quality of care

Next, we investigated the effects of the SAR intervention on our co-primary outcomes, namely, patient engagement and perceived quality of care. The results of these analyses are displayed in Table 3, whereas the effects on our secondary outcome are described in Section “Effects on health-related quality of life”.

**Table 3:** Patient engagement and perceived quality of care: changes from the baseline to post-intervention in the control and intervention group

	Baseline (T1)		Post-intervention (T2)		Comparison
	*M*	SD	*M*	SD	
*Patient engagement*					
Control group (*N* = 95)	4.31	0.63	4.20	0.67	*t* (94) = −1.39, *p* = 0.167, *d* = −0.14
Intervention group (*N* = 79)	4.32	0.54	4.34	0.47	*t* (78) = 0.31, *p* = 0.759, *d* = 0.04
*Perceived quality of care*					
Control group (*N* = 97)	6.33	0.92	5.89	1.30	*t* (96) = −2.86, *p* = 0.005^**^, *d* = −0.29
Intervention group (*N* = 77)	6.36	0.93	5.93	1.29	*t* (76) = −2.58, *p* = 0.012^*^, *d* = −0.29

*Notes*. ^*^ *p* < 0.050, ^**^ *p* < 0.010.

The results related to patient engagement showed a decrease in the control group, whereas the intervention group experienced a slight increase in patient engagement. However, the changes were not statistically significant in either of the two groups. Furthermore, we did not observe a statistically significant interaction, demonstrating that the changes in the intervention group were not statistically significantly different from the changes observed in the control group (*F*(1, 172) = 1.55, *p* = 0.215, *η*_p_^2^ = 0.01). Moreover, our results showed that the perceived quality of care decreased statistically significantly from the baseline to post-intervention in both the control and intervention groups. The trends of change were similar in both groups (*F*(1, 172) = 0.002, *p* = 0.966, *η*_p_^2^ = 0.00).

### Effects on health-related quality of life

Next, we investigated the changes in health-related quality of life from the baseline (T1) to post-intervention (T2). The results of these analyses are displayed in Table 4.

**Table 4:** Health-related quality of life: changes from the baseline to post-intervention in the control and intervention groups

	Baseline (T1)		Post-intervention (T2)		Comparison
	*M*	SD	*M*	SD	
*Mobility*					
Control group (*N* = 95)	2.69	0.46	2.69	0.46	*t* (94) = 0.00, *p* = 1.000, *d* = 0.00
Intervention group (*N* = 77)	2.70	0.46	2.74	0.47	*t* (76) = 0.62, *p* = 0.535, *d* = 0.07
*Self-care*					
Control group (*N* = 95)	2.91	0.29	2.76	0.43	*t* (94) = −3.73, *p* < 0.001^***^, *d* = −0.38
Intervention group (*N* = 77)	2.94	0.25	2.84	0.40	*t* (76) = −2.16, *p* = 0.034^*^, *d* = −0.25
*Usual activities*					
Control group (*N* = 95)	2.64	0.50	2.58	0.50	*t* (94) = −1.10, *p* =.0.276, *d* = −0.11
Intervention group (*N* = 78)	2.60	0.52	2.64	0.48	*t* (77) = 0.60, *p* = 0.552, *d* = 0.07
*Pain/discomfort*					
Control group (*N* = 95)	2.44	0.52	2.40	0.49	*t* (94) = −0.65, *p* = 0.519, *d* = −0.07
Intervention group (*N* = 77)	2.36	0.48	2.51	0.50	*t* (76) = 2.36, *p* = 0.021^*^, *d* = 0.27
*Anxiety/depression*					
Control group (*N* = 95)	2.61	0.55	2.78	0.42	*t* (94) = 3.62, *p* < 0.001^***^, *d* = 0.37
Intervention group (*N* = 78)	2.63	0.51	2.76	0.43	*t* (77) = 2.00, *p* = 0.049^*^, *d* = 0.23
*Whole scale (excluding VAS)*					
Control group (*N* = 95)	2.66	0.31	2.64	0.34	*t* (94) = −0.51, *p* = 0.610, *d* = −0.05
Intervention group (*N* = 77)	2.65	0.30	2.70	0.32	*t* (76) = 1.31, *p* = 0.195, *d* = 0.15
*Self-rated health (VAS)*					
Control group (*N* = 95)	68.05	17.91	71.31	20.36	*t* (94) = 1.52, *p* = 0.131, *d* = 0.16
Intervention group (*N* = 77)	69.42	18.57	73.12	17.25	*t* (76) = 1.58, *p* = 0.118, *d* = 0.18

The results showed that mobility, as one aspect of health-related quality of life, increased slightly in the intervention group, whereas it remained about the same in the control group; however, the changes were not statistically significant in either of the two groups. Similarly, we did not find significantly different patterns of change between the two groups (*F*(1, 170) = 0.23, *p* = 0.629, *η*_p_^2^ = 0.00). Next, the quality of life related to self-care decreased in both groups, but the change from T1 to T2 was significant only in the control group. The interaction was not statistically significant (*F*(1, 170) = 0.95, *p* = 0.332, *η*_p_^2^ = 0.01). The quality of life related to usual activities decreased slightly in the control group and increased slightly in the intervention group, but the changes were not statistically significant. The interaction was also not statistically significant (*F*(1, 171) = 1.39, *p* = 0.240, *η*_p_^2^ = 0.01), implying that the trends of change were relatively similar. Issues related to pain and discomfort increased in the control group, while they decreased in the intervention group, with the latter change being statistically significant. Moreover, the interaction was statistically significant as well, demonstrating different patterns of change between the two groups (*F*(1, 170) = 4.17, *p* = 0.043^*^, *η*_p_^2^ = 0.02). Issues related to anxiety and depression decreased in both groups from the baseline to post-intervention; the changes were statistically significant in both groups. However, the patterns of change were similar in both groups (*F*(1, 171) = 0.27, *p* = 0.605, *η*_p_^2^ = 0.00).

When we look at the results pertaining to the whole scale, we can notice that the score, interpreted as health-related quality of life decreased in the control group, whereas it increased in the intervention group. None of the changes were statistically significant, and the interaction was also not statistically significant (*F*(1, 170) = 1.76, *p* = 0.186, *η*_p_^2^ = 0.01). Lastly, self-rated health, measured with the VAS scale, increased slightly in both groups, but the changes were not statistically significant. Moreover, there was no interaction between the groups (*F*(1, 170) = 0.02, *p* = 0.888, *η*_p_^2^ = 0.00).

### Exploration of variables that moderate the effects of the intervention on the selected outcomes

**Fig. 2:**
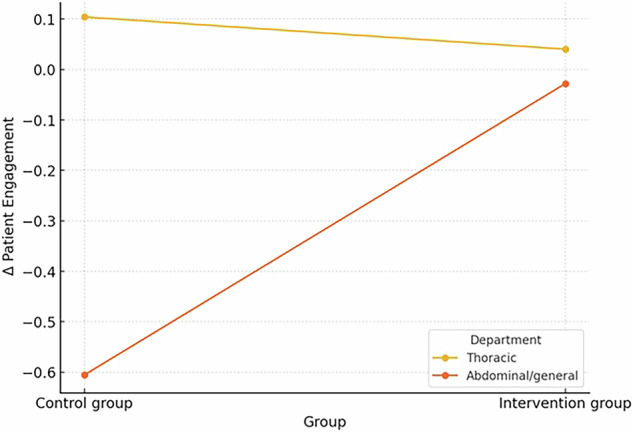
The statistically significant interaction between group and department. Moreover, the patients with a low acceptance of socially assistive robots (*b* = −0.08, *SE* = 0.15, *p* = 0.595) exhibited a slightly higher change in patient engagement if they were in the control group than the intervention group, but the difference was not statistically significant. On the other hand, the patients with a high acceptance of socially assistive robots (*b* = 0.30, *SE* = 0.13, *p* = 0.022^*^) exhibited a statistically significantly higher change in patient engagement if they were in the intervention group, compared to the control group. This significant interaction is visualized in Fig. 3.

**Fig. 3:**
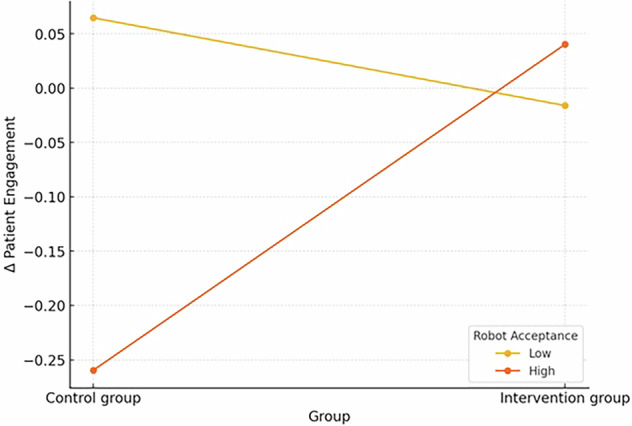
The statistically significant interaction between the group and robot acceptance. Similarly, we explored the potential moderators of the effects of the intervention on the perceived quality of medical care. We found that gender (*b* = 0.70, *SE* = 0.46, *p* = 0.129), age (*b* = 0.00, *SE* = 0.02, *p* = 0.827), education (*b* = 0.02, *SE* = 0.20, *p* = 0.915), department (*b* = 0.62, *SE* = 0.47, *p* = 0.184), and willingness to use the robot (*b* = 0.96, *SE* = 0.61, *p* = 0.114) were not significant moderators.

We first explored the potential moderators of the effects of the intervention on patient health engagement (PHE). We found that gender (*b* = 0.21, *SE* = 0.21, *p* = 0.335), age (*b* = 0.01, *SE* = 0.01, *p* = 0.334), and education (*b* = 0.07, *SE* = 0.09, *p* = 0.423) were not significant moderators, whereas individuals’ department (*b* = 0.64, *SE* = 0.22, *p* = 0.004^**^) and willingness to use the robot (*b* = 0.57, *SE* = 0.27, *p* = 0.039*) were. Specifically, in the case of thoracic surgery departments, the change in PHE from the baseline to post-intervention was similar in both groups (control and intervention; *b* = −0.06, *SE* = 0.12, *p* = 0.594). On the other hand, for the abdominal and general surgery patients, the negative change from the baseline to post-intervention was statistically significantly larger in the control group compared to the intervention group (*b* = 0.58, *SE* = 0.18, *p* = 0.002^**^). This significant interaction is visualized in Figs. 2 and 3.

Lastly, we explored the potential moderators of the effects of the intervention on health-related quality of life (separately for the whole scale, excluding VAS and the VAS score). For the whole scale, we found that gender (*b* = 0.04, *SE* = 0.10, *p* = 0.664), age (*b* = 0.00, *SE* = 0.00, *p* = 0.837), education (*b* = −0.05, SE = 0.04, *p* = 0.285), department (*b* = 0.14, SE = 0.10, *p* = 0.172), willingness to use the robot (*b* = 0.09, SE = 0.13, *p* = 0.505), change in PHE (*b* = 0.06, SE = 0.08, *p* = 0.425), and change in the perceived quality of medical care (*b* = −0.02, SE = 0.03, *p* = 0.469) were not significant moderators. Similarly, gender (*b* = 1.91, SE = 6.43, *p* = 0.767), age (*b* = 0.13, SE = 0.24, *p* = 0.600), education (*b* = −3.35, SE = 2.76, *p* = 0.226), department (*b* = 1.82, SE = 6.81, *p* = 0.790), willingness to use the robot (*b* = −2.76, SE = 8.31, *p* = 0.741), change in PHE (*b* = −1.12, SE = 4.91, *p* = 0.820), and change in the perceived quality of medical care (*b* = −2.31, SE = 2.17, *p* = 0.289) were not significant moderators of the VAS scores. The simple bivariate correlations between the changes in PHE, perceived quality of care, and health-related quality of life may be found in the Supplementary Information.

## Discussion

The evaluation presented in this study focused specifically on patient-centered outcomes to provide clear evidence regarding SAR intervention effects on patient experience and engagement. The feasibility of implementing the SAR intervention in surgical wards was demonstrated by the high retention rate and successful integration into existing care pathways. The demonstrated successful integration of the SAR with existing hospital care pathways addresses a limitation noted in previous research^36,37^, and demonstrates the potential for SAR technology to complement standard care practices in surgical settings. The study observed varying retention rates across different stages and groups. The retention rate from recruitment to the baseline was 100.0% (control) vs. 80.5% (intervention), while retention from the baseline to post-intervention was 90.1% (control) vs. 83.2% (intervention). This translates to dropout rates of 0.0% for control and 19.5% for the intervention group (recruitment to baseline), plus 9.9% for control and 16.8% for the intervention group (baseline to post-intervention). These retention rates align favorably with the typical dropout rates in clinical studies, which range from 5 to 40%.

The retention rate from the baseline to post-intervention in the intervention group was notably higher than the overall retention rate from initial recruitment, suggesting that most attrition occurred early in the study. The pattern of discomfort and poor tolerance leading to early withdrawal highlights the vulnerability of surgical patients to additional interventions during recovery periods. Postoperative pain, fatigue, and the cognitive effects of anesthesia may reduce the patients’ capacity to engage with novel technologies, regardless of their pre-surgical attitudes. The association of some refusals with worsening health status underscores the importance of monitoring the patient's clinical trajectory when implementing research interventions in acute care settings. The high rate of incomplete data reflects the additional assessment burden associated with technological components, indicating the need for streamlined data collection protocols in future SAR implementations. Administrative factors affecting both groups equally suggest that standard hospital operational challenges (transfers, rescheduling) contribute to attrition independently of intervention effects. No selective dropout was observed based on group allocation, gender, education, or age, which strengthens the validity of between-group comparisons^46^. However, a difference in dropout rates between the departments was noted, with abdominal and general surgery patients being more likely to discontinue participation compared to thoracic surgery patients, potentially due to different post-operative recovery trajectories or pain levels.

While any attrition in a study can be problematic, early attrition is generally considered more favorable, since participants who drop out early typically have not contributed much data, minimizing the amount of missing data in the analysis^47^. The early attrition observed in the intervention group is not ideal, but it is preferable to losing the same percentage later in the study^48^. It also suggests that the initial introduction to or requirements of the SAR intervention may have deterred some participants, which provides valuable information for refining future study protocols.

The impact of the SAR intervention on patient engagement revealed inconclusive results. While the overall changes in patient engagement were not statistically significant for either group, we observed a slight decrease in the control group and a marginal increase in the intervention group. The descriptive results align somewhat with the potential benefits of SARs in promoting patient engagement and self-management, as suggested by Chita-Tegmark & Scheutz^24^ and Robinson et al.^25^. However, it is worth noting that the observed changes were minor and not statistically significant. Moreover, additional exploratory analyses uncovered significant moderating effects. Patients in the abdominal and general surgery department showed a significantly larger negative change in engagement in the control group compared to the intervention group. The finding aligns with the observed attrition rates, where 20.5% of the abdominal and general surgery patients discontinued participation compared to only 9.0% of thoracic surgery patients. The consistency between lower engagement and higher dropout rates in this subgroup aligns with the previous stipulation on different post-operative recovery trajectories, where abdominal and general surgery patients may face unique challenges that affect both their willingness to continue participation and their overall engagement with their healthcare. This finding underscores further the complexity of patient engagement, as noted by Graffigna et al.^7^. Moreover, patients with high acceptance of SARs exhibited a statistically significant higher change in patient engagement if they were in the intervention group. These findings align with the technology acceptance model proposed by Broadbent et al.^36^, and suggest that the effectiveness of SAR interventions may be influenced by both contextual factors (e.g., type of surgery) and individual attitudes toward technology.

The results revealed a statistically significant decrease in both the control and intervention groups from the baseline to post-intervention. The similarity in trends between the groups suggests that the SAR intervention did not alter patients’ perceptions of quality of care compared to standard care significantly. As suggested by Cvetanovska et al.^15^, achieving active patient engagement can be challenging, particularly in complex healthcare environments such as surgical wards. The immediate post-operative period may be associated with discomfort, pain, and temporary limitations, which could impact patients’ perceptions of quality of care negatively, regardless of the interventions in place. Moreover, the introduction of innovative technology like a SAR might have raised patients’ expectations about quality of care. If these heightened expectations were not met fully, it could result in lower perceived quality of care, despite objective improvements in care delivery. The discrepancy between anticipated and actual experiences with the SAR might have contributed to the observed decrease in perceived quality of care Mlakar et al.^32^. This aligns with the findings of Christoforou et al.^27^, who noted that the integration of new technologies in healthcare settings can sometimes lead to initial dissatisfaction if not managed carefully. However, as stated earlier, a similar decline also occurred in the control group.

The effect of the SAR intervention on patients’ health-related quality of life varied across different dimensions. Notably, we observed a statistically significant interaction in the pain/discomfort dimension, with the intervention group showing a decrease in pain/discomfort issues, while the control group experienced an increase. This aligns with the potential of SARs to provide continuous support and education, as suggested by Barello et al.^5^ and Chita-Tegmark & Scheutz^24^, as well as to create pleasant and enjoyable interactions. For the overall health-related quality of life score, we observed a slight decrease in the control group and a minor increase in the intervention group, although these changes were not statistically significant. The lack of a significant interaction suggests that the SAR intervention did not have a substantial overall impact on health-related quality of life compared to standard care. This finding may be ascribed to health-related quality of life being a rather broad construct that is determined by a wide range of factors.

Our findings suggest that SARs may be most effective in specific, well-defined clinical domains rather than as broad-spectrum interventions^49^. The story-driven conversational capabilities were designed specifically to address the healthcare workforce shortages which result in time constraints that limit staff availability for extended patient education and non-urgent support. This creates an opportunity for SAR technology to fill these gaps without compromising the clinical care quality. The co-creative approach also addresses practical implementation concerns, as it allows healthcare institutions to integrate SAR technology gradually, without requiring changes to staffing models, clinical protocols, or regulatory frameworks. Furthermore, this approach aligns with the patient and staff acceptance patterns identified in our previous research, where the SAR technology was viewed most favorably when positioned as enhancing rather than replacing human care relationships^32^. While this design limits potential efficiency gains compared to substitutive models, it provides a more feasible and ethically sound foundation for initial SAR implementation in acute care settings.

Based on our results and observed patient interactions, high-potential applications include pain management support, where the statistically significant improvement in pain/discomfort management demonstrates the SARs’ ability to provide consistent, non-judgmental pain assessment and guided comfort interventions without nursing time constraints. Patient education and information delivery emerged as another strength, with high novelty scores and positive perspicuity ratings, indicating effective delivery of standardized discharge instructions, medication education, and procedure explanations. Additionally, the SARs showed particular value in managing non-urgent communication such as informational queries about meal times, visiting hours, and hospital navigation, that consume significant nursing time but require limited clinical judgment, as well as routine monitoring and data collection through standardized assessments like vital signs prompting and basic mobility checks. However, our findings suggest SARs are less suitable for complex emotional support, urgent medical decision-making, hands-on clinical procedures, and situations requiring nuanced clinical judgment or empathetic human connection, indicating that successful SAR implementation should focus on leveraging their strengths in structured, repeatable tasks, while preserving human care for complex interpersonal and clinical situations.

The effect sizes need to be interpreted in the context of the existing literature on SAR interventions in healthcare. Pu et al.^21^, in their systematic review and meta-analysis of SARs for older adults, found small to moderate effect sizes for various outcomes, including quality of life and psychological well-being. Overall, our findings, to some extent, align with this trend, suggesting that the impact of SAR interventions may be subtle but potentially meaningful, especially when considering the complex nature of healthcare environments.

In particular, for patient engagement, the effect size (Cohen’s *d*) was small for the intervention group. The small effect size for patient engagement may be noteworthy when considered alongside the work of Hibbard & Greene^9^, who emphasized the potential of patient activation to improve health outcomes and reduce healthcare costs. The moderate effect size for perceived quality of care), although negative, indicates that this outcome may be more sensitive to change, and could be a primary focus in future investigations. This finding aligns with the observations of Christoforou et al.^27^ and Mlakar et al.^32^ regarding the complex relationship between technology integration and perceived care quality. The small effect size for health-related quality of life is consistent with the findings of Kachouie et al. ^20^ in their mixed-method systematic literature review on SARs in elderly care.

While our study provides valuable insights into the implementation of SAR interventions in surgical wards, several limitations should be considered when interpreting the results and planning future research. Firstly, a significant limitation of our study stems from the integrated nature of our intervention, which combined patient-facing SAR technology with clinician-facing CDSS during ward rounds. While this design prevents us from isolating the individual contributions of the SAR versus CDSS components to the observed outcomes, due to complex intervention research^50^, it reflects the pragmatic reality of how digital health technologies are typically implemented in healthcare settings as integrated packages rather than isolated tools^27^. As a result, our analysis therefore focused on the overall effectiveness of this enhanced care model compared to standard care, which limits our ability to identify which specific technological components drove any observed effects. This design choice, while enhancing external validity and clinical relevance, reduced our capacity to provide targeted recommendations about individual intervention components, and may complicate replication efforts in settings where only partial implementation of the technology package is feasible. Future research should consider factorial designs or component dismantling studies, to understand the relative contributions of different digital health interventions better when implemented simultaneously. Moreover, the shift from a within-subject design to an RCT, while enhancing internal validity, introduced new variables and potential confounds. The transition to an RCT design likely increased the internal validity of our study, by reducing potential order effects and allowing for clearer between-group comparisons^51^. However, the change also necessitated a larger sample size, and potentially introduced new sources of variability between the groups. Finally, while not part of the original protocol, a dedicated study room was ensured for patients in the interventions to minimize spillover effects. It is worth noting that this is well in line with the CONSORT extension for non-pharmacological treatments^52^.

Second, the relatively short duration of our study may not have allowed sufficient time for staff and patients to acclimatize fully to, and leverage the benefits of, the SAR intervention. Future studies should consider longer intervention periods and follow-up periods to potential delayed effects of the intervention, and evaluate the potential (long-term) impacts that may emerge over time as users become more familiar with the technology. Namely, as suggested by Papadopoulos et al.^28^, the similar trends observed in both intervention and control groups for perceived quality of care might indicate that a longer adaptation period is necessary.

Third, our study may not have captured the complex, multifaceted nature of patient engagement and care quality fully, as highlighted by Krist et al.^10^ and Marzban et al.^11^. While the SAR intervention was innovative, it may not have addressed all the aspects that contribute to patients’ overall perception of quality of care. The lack of significant overall impact on health-related quality of life suggests that the benefits of SAR interventions may be more nuanced and domain-specific than initially anticipated. Our study found positive effects in specific areas, such as pain management, but these benefits did not translate to overall improvements in the health-related quality of life. This limitation underscores the need for more targeted applications of SAR technology, focusing on areas where it can provide the most substantial benefits. Moreover, while our overall retention rate was high, the observed differential attrition by department highlights the complexity of conducting research across diverse surgical populations. Moreover, the overall attrition can also be partially contributed to the co-primary endpoint design increasing patient assessment burden. However, the co-primary endpoints were snecessary to capture the multidimensional nature of SAR intervention effects. The alternative of selecting a single primary outcome would have provided an incomplete assessment of intervention effectiveness and potentially missed domain-specific benefits, as evidenced by our finding of significant pain management improvements that might not have been detected with a more limited outcome framework. Future studies should consider implementing targeted retention strategies for abdominal and general surgery patients and explore the factors contributing to higher dropout rates in this group. Moreover, future research should explore whether digital platforms integrated with SAR technology could reduce assessment burden by embedding outcome measurements within routine care interactions.

Fourth, the small to moderate effect sizes observed in our study, particularly for patient engagement and overall health-related quality of life, suggest that larger sample sizes may be needed to detect statistically significant effects. Linked to this, as highlighted in our discussion on the perceived quality of care, the introduction of innovative technology like a SAR might have raised patients’ expectations, potentially influencing their perceptions of quality of care. Our study may not have controlled for or measured these expectations adequately. Future research should consider assessing and managing patient and staff expectations as part of the study design. The presence of researchers and the novelty of the SAR intervention may have created a Hawthorne Effect, which influenced participant behavior, potentially affecting the study outcomes. Future studies should consider designs that minimize this potential bias, such as longer acclimatization periods or more naturalistic observation methods.

Fifth, our study lacks systematic integration between the technical performance data and clinical outcomes. While we collected technical metrics, including speech recognition accuracy, system response times, and navigation performance, we did not correlate these systematically with patient engagement, satisfaction, or dropout rates. This limited our ability to identify which technical factors influence clinical effectiveness most, and may have missed important mediating relationships between system performance and patient outcomes. Future studies should implement integrated monitoring systems that track technical performance and clinical responses simultaneously to optimize both technological development and patient care delivery. Moreover, while our study focused exclusively on the acute hospital period, the positive patient acceptance and demonstrated feasibility suggest potential for extending SAR interventions into post-discharge care. The transition from hospital to home represents a critical period, where patients must assume greater self-management responsibilities while having reduced access to professional support. Future research should investigate the feasibility and effectiveness of SAR interventions in post-discharge settings.

Finally, our clinical findings are derived exclusively from elective surgical patients in a controlled hospital environment, limiting their generalizability to other patient populations and healthcare settings. The planned nature of surgical care may have provided optimal conditions for demonstrating clinical benefits that might not translate to emergency, chronic care, or community-based contexts. Future research should examine clinical effectiveness across diverse patient populations and healthcare settings. Priority areas should include chronic disease management, rehabilitation settings, and outpatient care environments, where different patient characteristics and care needs may influence the clinical benefits observed in our surgical population.

To sum up, the study reported in this article demonstrates the feasibility of implementing SAR interventions in surgical wards and provides insights into their potential effects on patient outcomes. While we did not observe significant overall effects on all the measured outcomes, the identified moderating factors and the significant interaction in pain/discomfort management highlight promising avenues for future research. Our findings thus contribute to the growing body of literature on SAR applications in healthcare^16,17^, and address the gap in research on SAR use in surgical settings. They also align with the call for more robust, context-specific studies on SAR implementation in healthcare settings^18^. The successful technical implementation, combined with the clinical feasibility and preliminary effectiveness shown in the present study, provides a foundation for future larger-scale trials, which should integrate technical performance monitoring with clinical outcome assessment systematically.

Beyond the primary objectives, the potential implications to clinical practice are broader. The successful implementation and high retention rates demonstrate that SAR technology can feasibly be integrated into existing care pathways. This suggests that healthcare institutions can consider a SAR as a viable option for augmenting care delivery, potentially addressing some of the challenges related to workforce shortages^1,2^. However, the differential effects observed across surgical departments highlight the need for tailored approaches with department-specific factors, rather than adopting a one-size-fits-all approach. Furthermore, the potential of a SAR to provide continuous support and education aligns with the growing emphasis on patient self-management^5,6^. Healthcare providers might leverage a SAR for consistent delivery of patient education and support, particularly during periods when staff availability is limited. Moreover, the significant positive impact on pain/discomfort management suggests that a SAR could be particularly useful in the aspect of post-operative care. Healthcare providers might explore targeted applications of a SAR for pain management protocols. Finally, the study results suggest that a SAR should be viewed as a complement to, rather than a replacement for, human care. Clinical practice should focus on integrating a SAR in ways that enhance, rather than substitute, human-delivered care. Overall, our findings may be most generalizable to patient populations with similar characteristics, e.g., planned medical procedures (cardiac catheterizations, endoscopies, outpatient surgeries), structured rehabilitation settings (physical therapy, post-stroke recovery), and chronic disease management requiring regular monitoring and education. The SAR’s strengths in providing consistent education, routine assessment, and non-urgent communication support may translate well to these contexts.

## Methods

### Study design

**Fig. 4:**
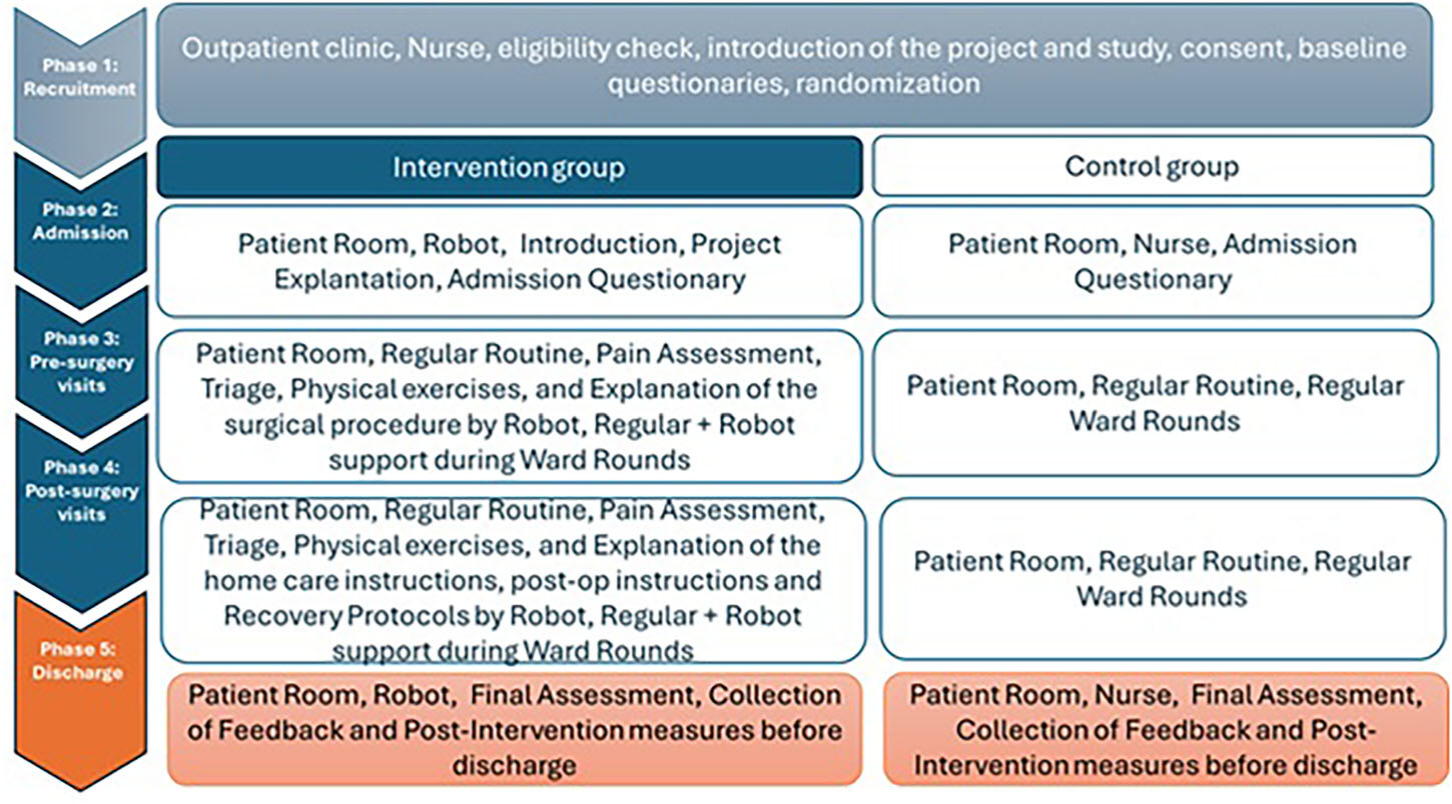
Overview of the study design. All the patients in both groups received identical standard nursing care, medical care, and physiotherapy from human staff according to the usual hospital protocols. The intervention group was additionally exposed to interactions with a SAR, Frida^62^, during various stages of their hospital stay. The SAR provided additional educational content, support, and interaction opportunities that would not otherwise be available due to staff time constraints, rather than substituting for existing human-delivered care. As outlined in Fig. 1, the Recruitment (phase 1) was carried out at the outpatient clinic. The patients were first screened against inclusion/exclusion criteria (see section “Participants”). If they were eligible, they were informed about the study by a dedicated recruiter and asked to sign a written letter of consent. The participants in both groups completed baseline measures upon Recruitment (phase 1) and post-intervention measures just before discharge (phase 5).

This study was carried out as a randomized controlled study design with two arms: an intervention group and a control group, with a 1:1 allocation. The external pilot trial study was registered under the ISRCTN Registry (ISRCTN96689284). It was conducted in the thoracic surgery and abdominal and general surgery wards of the University Clinical Center Maribor, Slovenia, between June 2023 and May 2024. Patients admitted for elective procedures were allocated randomly to either group. No long-term follow-up was planned beyond hospital discharge. The participants completed outcome measures at baseline (upon recruitment) and post-intervention (just before discharge). The study design is outlined in Fig. 4.

During admission (phase 2), control group filled in the regular admission questionnaire in paper format, and the SAR greeted patients in the intervention group, explained and presented the project, and offered an interactive form of filling in the admission questionnaire, through speech-enabled and SAR-moderated interaction. Additionally, during ward rounds, the intervention group patients had their cases reviewed with optional use of the CDSS delivered via the SAR, while the control group patients had their cases discussed during standard ward rounds procedures without technological augmentation. It is worth noting that ward rounds refer to formal clinical meetings where healthcare teams discuss patient cases, review treatment plans, and make clinical decisions. In our study context, these were regularly scheduled ward meetings, where the medical team reviewed patient progress and made care decisions. During these, the CDSS component (i.e., the electronic system providing clinicians with patient-specific assessments) was used to display patient information and trigger alerts for a redefined set of clinical outcomes. In both phase 3 and phase 4, the key difference between the intervention and control groups was the use of the SAR to deliver information, conduct assessments, and provide guidance. For the intervention group, the pre-surgery visits (phase 3) and post-surgery visits (phase 4) consisted of daily interactions with the SAR in a dedicated intervention room.

The data collection and outcome assessments were conducted by researchers not involved in patient clinical care to minimize potential conflicts of interest. The randomization was performed using the digital coin toss method in the outpatient clinic, before admission to the ward. The researcher supervised SAR interactions during the intervention, but did not conduct outcome assessments or analysis to reduce bias. Standard clinical care was provided by ward nursing and medical staff, who were aware of the patient group allocation, but were instructed to maintain the usual care protocols for all the participants regardless of the group assignment.

### Changes to the original design

A significant modification was made to the study methods before the pilot trial study commencement, driven by pragmatic considerations and reflecting the realities of implementing research in a clinical setting. Namely, we combined the present patient-facing SAR study (registered under ISRCTN96689284) with a complementary clinician-facing study on clinical decision support systems (CDSS) during ward rounds (registered under ISRCTN12048782). This combination was motivated by time and resource constraints, the co-creation workshops, and, more importantly, the recognition that these interventions operate synergistically in real-world healthcare settings. Namely, patient outcomes are influenced both by direct patient engagement tools (SAR) and by the quality of clinical decision-making (CDSS). Evaluating these components together provides a more ecologically valid assessment of comprehensive digital health implementation. This resulted in patients in the intervention group receiving direct SAR interactions, while also benefiting indirectly from enhanced clinical decision-making when the CDSS was utilized during their care. Importantly, this did not create a dual intervention burden for patients, as the CDSS operated at the clinician level during the ward rounds and care planning, while the SAR provided direct patient-facing support. Moreover, the CDSS component required consistent implementation during the ward rounds across the study period, making crossover designs impractical for the clinical team. Thus, in addition to delivering two complementary interventions, we have also modified the originally planned within-subject design (*N* = 90) to a more robust RCT design with the larger sample sizes.

### Participants

Those admitted to thoracic and abdominal/general surgery wards for an elective (non-emergency) procedure were screened for eligibility regarding the inclusion and exclusion criteria. Specifically, beyond the department they were admitted to, the patients needed to be aged 18 years or above, willing to participate in the study, and willing and eligible to sign the informed consent. On the other hand, the exclusion criteria included emergency patients, patients already enrolled in other studies, patients with dementia, special needs, incapable of verbal communication or appointed guardians, and patients allocated to an intensive step-down unit or regimen.

The eligible patients were allocated to the control group using a simple coin toss method using the Google Coin Flip software. Heads meant that the patient was assigned to the intervention group (SAR intervention), and tails that the patient was assigned to the control group (standard care). A research assistant not involved in patient care or outcome assessment performed the digital coin toss at the time of each participant’s enrollment, ensuring immediate and unbiased allocation. After randomization, the patients entered the study immediately.

The final sample size of 200 was determined by the power requirements of the combined evaluation framework, providing adequate power to detect meaningful effects on our co-primary patient-centered outcomes. Specifically, the requirements of the CDSS evaluation component were, based on a power analysis for detecting clinically meaningful differences in health quality measures, determined at *N* = 200. Moreover, the SAR intervention component, originally planned for 90 participants in a within-subject design, was integrated into this larger RCT framework. The power analysis for the combined study confirmed that *N* = 200 provided adequate power (1-β = 0.80, *α* = 0.05) to detect small-medium effect sizes (*f* = 0.15) for patient engagement and perceived quality of care outcomes using a mixed-design ANOVA.

The co-primary endpoint design accounts for potential correlation between patient engagement and perceived quality of care, which enhances rather than compromises statistical efficiency. Recent methodological advances demonstrate that consideration of correlation between co-primary endpoints can increase trial power and reduce required sample size requirements^53^. No multiplicity adjustment is required for co-primary endpoints since significance must be achieved on both outcomes, inherently controlling Type I error and reducing the chance of erroneous efficacy conclusions compared to single endpoint designs.

### Blinding procedures

Complete blinding of the participants was not feasible due to the nature of the SAR intervention. Namely, the patients could distinguish clearly between receiving robot-delivered education/support versus standard care only. However, the control group participants were not informed about the specific content or extent of the SAR intervention to minimize the expectation bias.

The researchers evaluating the outcomes and carrying out the statistical analyses were not blinded to the group allocation, which represents a limitation of our study design. However, all the outcome measures were patient-reported questionnaires completed independently by the participants, which mitigated assessor bias risk somewhat.

### Ethics and consent

For the study, a separate ethics approval was received from the Medical Ethics Commission of the University Medical Center Maribor, Ljubljanska ulica 5, 2000 Maribor, Slovenia; (+386 (0)2 321 2489; eticna.komisija@ukc-mb.si), on 15 December 2021, registration number: UKC-MB-KME-76/21. Informed consent was obtained from all the subjects involved in the study in paper format. The patients and staff in the images signed a written consent to participate in the study. The consent included publication of the images.

### Measures and outcomes

All the participants were asked to fill out the baseline measure (demographic variables, willingness to use the robot, patient engagement, perceived quality of medical care, and health-related quality of life) and the follow-up measure that occurred post-intervention (patient engagement, perceived quality of medical care, and health-related quality of life). All the scales previously unavailable in Slovene were translated using the translation-backtranslation procedure. The internal reliabilities of all the questionnaires used in this study are reported in the Results section.

### Demographic variables

At the beginning of the baseline measure, the participants were asked about their gender (female, male, other), age (in years), and highest education level (primary school or less, lower secondary education, higher secondary education, Bachelor’s degree or equivalent, Master’s degree or equivalent, Doctoral degree or equivalent). We also recorded the surgery department to which they were admitted (i.e., either the thoracic surgery department or the abdominal and general surgery department).

### Primary outcomes

Patient engagement and perceived quality of care were selected as the co-primary outcomes, based on their direct relevance to SAR intervention mechanisms and established prognostic value in healthcare settings. For patient engagement, we used the PHE scale^7^, a widely used measure of patient engagement, available in several languages and suitable for various patient subgroups. The scale consists of nine items (e.g., »Despite my illness, I know how to manage my life«), which were answered on a 5-point agreement scale by the patients in our study (1 – »Completely disagree«, 5 – »Completely agree«). Score interpretation follows established benchmarks: scores ≤3.0 indicate low engagement, 3.1-4.0 moderate engagement, and >4.0 high engagement levels. The PHE scale demonstrates established concurrent validity with the patient activation measure (*r* = 0.431, *p* < 0.001) and shows sensitivity to treatment-related changes in patient engagement interventions. Test-retest reliability using ICC yielded excellent results after 15 days (ICC = 0.95; CI = 0.90−0.97). In the Supplementary Information, we also report the results obtained using only five items of the scale that constituted the short version used commonly in previous research. In the original validation study, both versions of the scale exhibited good internal consistency (*α* = 0.97)^7^.

To measure the perceived quality of care, we used the Perceived Quality of Medical Care (PQMC) scale^54^. The scale has six items, answered using a 7-point semantic differential scale. An example item is “The medical care I have received was unsatisfactory/satisfactory”. The scale has one dimension. Four of the six items (items 1, 2, 4, and 5) were recoded so that higher average scores indicated higher perceived quality of care. In the original validation study, the scale exhibited excellent internal consistency (*α* = 0.94; 39). Beyond the validation study, the scale has previously been used to investigate several factors that contribute to the perceived quality of care^55,56^. The original validation study demonstrated convergent validity of the PQMC through significant negative correlations with communication apprehension (r = –0.45, *p* < 0.01), indicating that higher perceived quality of care is associated with lower patient anxiety about communicating.

### Other measures

Health-related quality of life was designated as the secondary outcome, to capture the broader impacts of the intervention on patient well-being. To assess health-related quality of life we used the EQ-5D-3L instrument^57^. The scale consists of six items. The first five items correspond with the five dimensions of health-related quality of life: mobility, self-care, usual activities, pain/discomfort, and anxiety/depression. Each item has three response levels: no problems, some problems, and extreme problems. We recoded the items so that the higher scores indicated a higher health-related quality of life. The items can be analyzed separately or converted into a single index value using standardized scoring algorithms. Previous studies with older adult patients demonstrated acceptable internal consistency of the dimensions (α = .67)^58^. Additionally, the sixth item of the scale is essentially a visual analog scale (VAS), that records the patient’s self-rated health from 0 (the worst health one can imagine) to 100 (the best health one can imagine). The Slovenian version of the scale was validated by Prevolnik–Rupel and colleagues^59^. Lastly, individuals’ acceptance of SARs in a hospital environment was measured with four questions (e.g., “Would you like to be treated in a hospital where a robot is part of the medical team?”), previously developed by the authors. The questions were answered by using a dichotomous response format (“No” or “Yes”). The participants’ answers were then averaged, with higher values indicating a higher acceptance of SARs. In our previous research^32^, the scale exhibited good internal consistency (*α* = 0.82).

### Co-creation workshops for study and intervention design

The design of this pilot study, including the selection of co-primary outcomes and the intervention itself, was informed by a systematic co-creation process conducted during the HosmartAI project development phase. This process utilized a Living Lab methodology with iterative extended sprints, as detailed by Arioz et al.^38^. Sprint 1 involved a comprehensive stakeholder workshop with 24 participants representing three key groups: researchers, nurses, and project team members. This phase focused on understanding the problem space, identifying expected functionalities of the SAR, and exploring implementation limitations within hospital workflow. Through collaborative discussions, medical doctors and nurses identified suitable tasks for the robot and discussed potential challenges in integrating robotic assistance into patient care. Stakeholders emphasized the importance of measuring both patient participation (engagement) and subjective care evaluation (perceived quality) as complementary dimensions necessary to evaluate SAR effectiveness. Sprint 2 continued the co-creation process through four face-to-face workshops involving 51 participants from diverse stakeholder groups, including researchers, nurses, medical doctors, physiotherapists, and project coordinators. These workshops systematically addressed: (1) finalizing the robot’s architecture and placement in the real-world clinical environment, (2) defining the conceptual design for human-robot interaction, (3) designing care plans and digital interventions, and (4) identifying and validating deployment scenarios and outcomes measured. Participants interacted directly with the robot (named Frida), fostering familiarity and gauging its potential appeal to patients, with a positive reception serving as a promising indicator for future implementation. The workshops further confirmed stakeholder consensus that successful SAR implementation requires demonstrating both active patient participation (engagement) and positive subjective care evaluation (perceived quality).

### Intervention

**Fig. 5:**
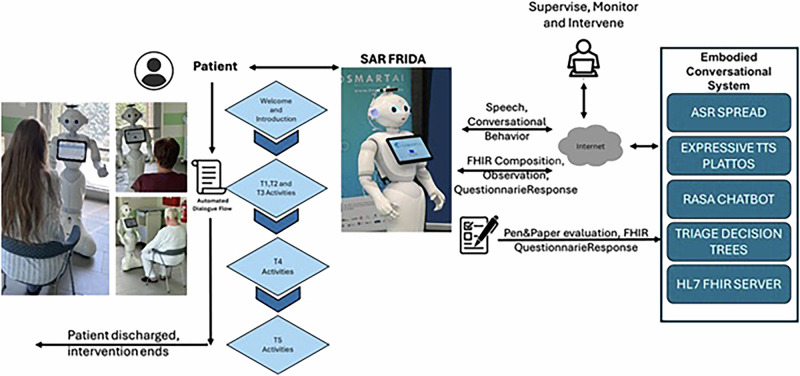
Implementation and delivery of the digital intervention. The SAR intervention was designed as complementary to standard care rather than replacing human staff functions, aligning with current best practices in healthcare technology implementation and ethical considerations around patient safety^27–29^. To ensure response accuracy and clinical appropriateness, all the SAR dialog content underwent systematic validation (see Table 5). The clinical staff, including nurses, physicians, and physiotherapists, were facilitated in the design/definition phase, but also reviewed and approved all the programmed responses during the system development phase. This ensured that all the critical clinical functions remained under human oversight, while leveraging the SAR’s unique capabilities for consistent, available-on-demand educational support and interaction.

The intervention utilized the Pepper robot (SoftBank Robotics), a 120 cm tall humanoid robot known for its affordability and popularity in human-robot interaction studies^60^. However, recognizing Pepper’s limitations in advanced communicative capabilities and support for non-mainstream languages^61^, significant customizations were made to optimize its performance for this study. The SAR intervention utilized a decentralized symmetric interaction. The details on the technical solution, outlined in Fig. 5, are described in detail in ref. ^62^ and the results, related to evaluation of the technical components, are summarized in *Supplementary Information (Result of evaluation of Technical Components)*. The center of these enhancements for the Pepper Model was the integration of an advanced Embodied Conversational System. The system was comprised of several elements working together to deliver a seamless and effective interaction between the robot and the patients. We deployed an RASA-based Chatbot^63^ to orchestrate structured interactions between the patients and the SAR. Unlike large language models, the system employed a deterministic, rule-based approach, where each clinical activity was designed as a white-box model with well-defined story lines and finite inputs/outputs. Each patient’s interaction scenario (pain assessment, physical exercises, discharge instructions, basic triage) was designed as a structured “story line” with finite, predetermined inputs and outputs that were co-designed and validated by nurses, clinicians, and patients during the co-creation phase of the EU-funded HosmartAI project^38^. RASA’s story-driven methodology defined a set of chatbot behaviors through predefined conversation examples that outlined the expected dialog flows for each clinical scenario. This white-box design prioritized transparency, safety, and clinical accuracy over conversational flexibility, ensuring that all the responses could be validated by healthcare professionals and aligned with hospital protocols. The PLATTOS speech synthesis system was implemented to overcome Pepper’s limitations in speech production, especially in the Slovenian language. This end-to-end multilingual and multimodal text-to-conversation synthesis system utilized a multi-stage architecture, which included speech production and a gesture synthesis system. The ASR SPREAD (Automatic Speech Recognition) system was integrated to enable the robot to understand and respond to the patients’ verbal inputs. A critical aspect of the intervention’s design was its integration with standardized healthcare data systems, specifically through the implementation of FHIR (Fast Healthcare Interoperability Resources). The study utilized an HAPI FHIR v2 based infrastructure, focusing on key FHIR resources such as *Patient*, *Questionnaire Response*, and *Composition*. This integration allowed for all the patient responses and interactions to be stored in a standardized, interoperable format.

Throughout the process, as outlined in Fig. 2, the SAR also delivered gamified nursing education, providing critical information on wound care, home care considerations, and daily care explanations through engaging, multimodal interactions. The physiotherapy guidance component utilized both verbal instructions and physical demonstrations, leveraging the robot’s capability for movement and gesture synthesis. The system also offered ongoing patient assistance for non-urgent communication and support, with all the interactions designed carefully to work within the enhanced capabilities of the Pepper platform. All the engagements were supervised by an experimenter, whose role was to start the experiment by inserting a patient ID and initiating the dialog flow. During the process, the experimenter observed and intervened if errors occurred in the discourse flow.

### Statistical analysis

We used the IBM SPSS Statistics 28 software for the statistical analysis. We first cleaned the dataset, and excluded all the participants who did not provide us with their demographic data and at least partly filled out the baseline measure. We continued with other preparatory steps. First, we performed tests of initial equivalence and selective dropout. Second, we investigated the internal consistencies of all multiple-item scales using the coefficient α, and calculated the factor scores in accordance with the scoring instructions. Third, we checked the assumptions of the chosen statistical tests (such as the normality of the distribution assumption).

Next, we calculated the bivariate correlations between all the variables of interest and conducted various inferential tests to investigate our research questions and hypotheses. Changes from the baseline to post-intervention within each group (control, intervention) were investigated using paired samples *t*-tests. The main analyses, in which we investigated the relative effectiveness of the intervention (standard care with robot interactions) compared to the control group (standard care) on the selected outcomes, were performed using separate mixed-design (split-plot) analyses of variance (ANOVA), with the allocated group as the between-subjects factor, and time as the within-subjects factor. Additional exploratory analyses, in which we investigated the potential moderators that attenuate or accentuate the effects of the intervention, were conducted using the PROCESS MACRO extension for SPSS^64^. Using Model 1, group allocation was used as the predictor variable, gender, age, education, department, and acceptance of robots as moderator variables, and the change (T2–T1) in patient engagement, perceived quality of care, and health-related quality of life as the outcomes. Additionally, the change in patient engagement and perceived quality of care were explored as moderators of the change in the health-related quality of life as well. The results were interpreted as statistically significant if the *p*-value was below 0.05. All the results were accompanied by effect sizes, such as Cohen’s *d*.

**Table 5:** Validated story-driven conversational capabilities (i.e., the automated dialog flows)

Component	Validation method	Clinical oversight
Collection of common symptoms,	Nurse and physician review	Real-time supervision
Exercise instructions	Physiotherapist validation	Demonstration verification
Triage/Alert decision trees	Nurse and physician co-design	Escalation protocol testing, Real-time supervision
Information about surgical procedures and pre/post procedure activities	Defined and designed by physicians	Content, Demonstration verification
Hospital Information (schedules, visitation hours, etc.)	Nursing and administrative staff review	Content accuracy verification
Discharge information	Clinical team approval	Content accuracy verification

## Supplementary information

Supplementary Information

## Data Availability

The datasets used and/or analyzed during the current study are available from the corresponding author upon reasonable request.

## Code availability

The underlying code for this study [and training/validation datasets] is not publicly available, but may be made available to qualified researchers upon reasonable request from the corresponding author.
