# Supplementary material for: A randomized pilot study evaluating socially assistive robot effects on patient engagement and care quality
Source: NPJ Digit Med. 2025 Dec 2;8:738. doi: 10.1038/s41746-025-02117-9 (PMC12672561; doi:10.1038/s41746-025-02117-9)
Supplement: Supplementary file 1 — Supplementary Information [file 41746_2025_2117_MOESM1_ESM.pdf]

**Supplementary Information**

1. *Patient Health Engagement: Results for the short version of the scale*

The short version of the PHE scale exhibited great internal reliability at the baseline (α = .87) and post-intervention (α = .88).

Similarly to the results obtained using the whole scale, the change from T1 to T2 was not statistically significant in the control group (T1: *M* = 4.21, *SD* = 0.71; T2: *M* = 4.19, *SD* = 0.71; *t*(94) = -0.26, *p* = .795, *d* = -0.03) nor the intervention group (T1: *M* = 4.23, *SD* = 0.61; T2: *M* = 4.32, *SD* = 0.49; *t*(78) = 1.18, *p* = .243, *d* = 0.13). The interaction was, again, not statistically significant ($F(1, 172) = 0.88$, $p = .349$, $\eta_p^2 = 0.01$).

*Supplementary Table 1*

*Correlations between the changes in patient health engagement, perceived quality of care, and health-related quality of life*

| | *N* | *M* | *SD* | 1 | 2 | 3 | 4 | 5 | 6 | 7 | 8 |
|---|---|---|---|---|---|---|---|---|---|---|---|
| 1. Gender | 206 | 1.43 | 0.50 | - | | | | | | | |
| 2. Age | 206 | 61.20 | 13.80 | .11 | - | | | | | | |
| 3. Education | 206 | 2.81 | 1.20 | -.11 | -.26$^{***}$ | - | | | | | |
| 4. Department | 206 | 1.35 | 0.48 | .11 | -.17$^{*}$ | .15$^{*}$ | - | | | | |
| 5. Acceptance of robots | 172 | 0.72 | 0.39 | .05 | -.07 | .13 | .15 | - | | | |
| 6. Δ Patient engagement | 174 | -0.05 | 0.70 | -.06 | -.05 | -.02 | -.28$^{***}$ | -.13 | - | | |
| 7. Δ Perceived quality of care | 174 | -0.43 | 1.48 | .01 | -.04 | .00 | -.28$^{***}$ | -.04 | .43$^{***}$ | - | |
| 8. Δ Health-related quality of life | 172 | 0.01 | 0.33 | -.01 | .08 | -.13 | -.30$^{***}$ | -.08 | .24$^{**}$ | .23$^{**}$ | - |
| 9. Δ Self-rated health | 172 | 3.45 | 20.63 | -.02 | .08 | .00 | -.16$^{*}$ | .05 | .17* | .14 | .26$^{***}$ |

2. *Result of evaluation of Technical Components*

The SAR intervention utilized a decentralized symmetric interaction model with the technical capabilities detailed in our parallel technical evaluation[45]. The technical performance of the system is summarized in *Supplementary Table 2.*

*Supplementary Table 2*

*System's technical performance*

| **Technical Component** | **Performance Metric** | **Clinical Relevance** |
|---|---|---|
| **Speech Recognition (SPREAD ASR)** | 5.71% WER on test data | Accurate patient communication |
| **Text-to-Speech (PLATTOS)** | MUSHRA scores 77.49-81.56 | Natural, acceptable voice quality |

| | | |
|---|---|---|
| **Navigation (ORB-SLAM2)** | >95% autonomous success rate | Reliable patient room access |
| **System Response Time** | 2-3 seconds average | Acceptable interaction flow |
| **Network Performance** | 40-60ms latency | Real-time communication capability |

The key performance metrics relevant to the clinical implementation included: speech recognition accuracy of 5.71%, word error rate on weal world data using the SPREAD ASR system, text-to-speech quality scores of 77.49-81.56 on the MUSHRA evaluation scales, and autonomous navigation success rates >95% using offloaded ORB-SLAM2 implementation. The system response latency averaged 40-60ms network delay plus 2-3 seconds processing time, which pilot testing indicated was acceptable to patients.

Despite robust laboratory performance, the real-world hospital deployment revealed several operational challenges. The SPREAD ASR system struggled occasionally with patient speech variations, due to post-operative conditions including sedation effects, pain-related speech changes, and individual dialect variations, that sometimes required multiple recognition attempts. The background noise in the hospital environment interfered periodically with the speech processing, requiring system recalibration. Navigation challenges included occasional difficulties with the ORB-SLAM2 system in hospital corridors with limited visual features, though autonomous navigation success rates remained >95% overall.

All the SAR interactions were supervised by trained research staff, whose role included initiating patient sessions and intervening when technical errors occurred in the dialog flow. Observational data, as reported in Mlakar et al. 2025[45], indicated that technical issues requiring human intervention were manageable, and did not appear to impact the patient satisfaction scores significantly, though some patients experienced initial difficulty with the conversational flow, particularly older adults less familiar with digital interfaces. Namely, when technical issues arose, including occasional dialog loop errors, the supervised implementation model allowed for real-time problem resolution without significant disruption to patient care. Despite these technical challenges, the overall technical implementation demonstrated feasibility for hospital deployment. The system's ability to handle routine patient interactions, conduct basic triage through decision-tree algorithms, and maintain transparent activity logging via blockchain technology, proved suitable for the clinical environment.

*3. CONSORT 2010 checklist*

*Supplementary Table 3:*

*CONSORT 2010 checklist of information to include when reporting a pilot or feasibility trial*

| Section/Topic | Item No | Checklist item | Reported on page No |
|---|---|---|---|
| **Title and abstract** | | | |
| | 1a | Identification as a pilot or feasibility randomised trial in the title | YES (p1) |
| | 1b | Structured summary of pilot trial design, methods, results, and conclusions (for specific guidance see CONSORT abstract extension for pilot trials) | YES (p2) |
| **Introduction** | | | |
| Background and objectives | 2a | Scientific background and explanation of rationale for future definitive trial, and reasons for randomised pilot trial | YES (p2, p3) |
| | 2b | Specific objectives or research questions for pilot trial | YES (p4) |
| **Methods** | | | |
| Trial design | 3a | Description of pilot trial design (such as parallel, factorial) including allocation ratio | YES (p18) |
| | 3b | Important changes to methods after pilot trial commencement (such as eligibility criteria), with reasons | YES (p20) |
| Participants | 4a | Eligibility criteria for participants | YES (p20-21) |
| | 4b | Settings and locations where the data were collected | YES (p20-21) |
| | 4c | How participants were identified and consented | YES (p21) |
| Interventions | 5 | The interventions for each group with sufficient details to allow replication, including how and when they were actually administered | YES (p22-24) |
| Outcomes | 6a | Completely defined prespecified assessments or measurements to address each pilot trial objective specified in 2b, including how and when they were assessed | YES (p21-22) |
| | 6b | Any changes to pilot trial assessments or measurements after the pilot trial commenced, with reasons | YES (p20) |
| | 6c | If applicable, prespecified criteria used to judge whether, or how, to proceed with future definitive trial | N/A |
| Sample size | 7a | Rationale for numbers in the pilot trial | YES (p20) |

| | | | |
|---|---|---|---|
| | 7b | When applicable, explanation of any interim analyses and stopping guidelines | N/A |
| Randomisation: | | | |
| Sequence generation | 8a | Method used to generate the random allocation sequence | YES (p21) |
| | 8b | Type of randomisation(s); details of any restriction (such as blocking and block size) | N/A |
| Allocation concealment mechanism | 9 | Mechanism used to implement the random allocation sequence (such as sequentially numbered containers), describing any steps taken to conceal the sequence until interventions were assigned | N/A |
| Implementation | 10 | Who generated the random allocation sequence, who enrolled participants, and who assigned participants to interventions | YES (p21) |
| Blinding | 11a | If done, who was blinded after assignment to interventions (for example, participants, care providers, those assessing outcomes) and how | YES (p21) |
| | 11b | If relevant, description of the similarity of interventions | N/A |
| Statistical methods | 12 | Methods used to address each pilot trial objective whether qualitative or quantitative | YES (24-25) |
| **Results** | | | |
| Participant flow (a diagram is strongly recommended) | 13a | For each group, the numbers of participants who were approached and/or assessed for eligibility, randomly assigned, received intended treatment, and were assessed for each objective | YES (p6) |
| | 13b | For each group, losses and exclusions after randomisation, together with reasons | YES (p6) |
| Recruitment | 14a | Dates defining the periods of recruitment and follow-up | N/A |
| | 14b | Why the pilot trial ended or was stopped | N/A |
| Baseline data | 15 | A table showing baseline demographic and clinical characteristics for each group | YES (p5) |
| Numbers analysed | 16 | For each objective, number of participants (denominator) included in each analysis. If relevant, these numbers should be by randomised group | YES (p5) |
| Outcomes and estimation | 17 | For each objective, results including expressions of uncertainty (such as 95% confidence interval) for any estimates. If relevant, these results should be by randomised group | YES (p7-p9) |

| | | | |
|---|---|---|---|
| Ancillary analyses | 18 | Results of any other analyses performed that could be used to inform the future definitive trial | YES (p6, p8) |
| Harms | 19 | All important harms or unintended effects in each group (for specific guidance see CONSORT for harms) | YES (p7) |
| | 19a | If relevant, other important unintended consequences | N/A |
| **Discussion** | | | |
| Limitations | 20 | Pilot trial limitations, addressing sources of potential bias and remaining uncertainty about feasibility | YES(p16, p17) |
| Generalisability | 21 | Generalisability (applicability) of pilot trial methods and findings to future definitive trial and other studies | YES(p17, 18) |
| Interpretation | 22 | Interpretation consistent with pilot trial objectives and findings, balancing potential benefits and harms, and considering other relevant evidence | YES(13 – 15) |
| | 22a | Implications for progression from pilot to future definitive trial, including any proposed amendments | YES(p17, 18) |
| **Other information** | | | |
| Registration | 23 | Registration number for pilot trial and name of trial registry | YES(p18) |
| Protocol | 24 | Where the pilot trial protocol can be accessed, if available | N/A |
| Funding | 25 | Sources of funding and other support (such as supply of drugs), role of funders | VIA SUBMISSION |
| | 26 | Ethical approval or approval by research review committee, confirmed with reference number | YES (p21) |

Note: Citation - Eldridge SM, Chan CL, Campbell MJ, Bond CM, Hopewell S, Thabane L, et al. CONSORT 2010 statement: extension to randomised pilot and feasibility trials. BMJ. 2016;355.
